# A Hospital Garden

**Published:** 1890-12-20

**Authors:** 


### A Hospital Garden.

If it were possible for each one of our metropolitan hospitals to stand surrounded by a veritable garden, how happy and beneficial would be the conjunction. But the home of the hospital is not unfrequently a crowded neighbourhood in whose gloomy atmosphere not one fair blossom could be tempted to unfold, and the flowers which are among the necessaries of hospital life, and are nowhere so highly prized or so warmly welcomed as in the hospital ward, must be brought from afar. Their value in the sick room is now very generally recognised. Many graceful gifts are made by country and suburban friends to most of the London hospitals, and in this way a really bountiful supply is kept up during the spring and summer months.

But there are flowers and flowers. Not a few which look pretty enough while growing, are but poor limp weedy things after they have been cut and packed, or they are too fragile to bear the journey. Even roses—and who would not welcome roses?—are apt to fall to pieces, though they be handled with the greatest tenderness, and the same must be said of single geraniums, pelargoniums, peonies, calceolarias, and a host of other flowers commonly despatched, which by the time they reach their journey's end have half filled the basket with loose petals.

What is wanted in cut flowers is substantiality. This means pre-eminently tenacity of life and ability to retain colour, form, and—if the blossom be so endowed—fragrance. Length and strength of stalk are also desirable. It may be said, and not ungratefully, that the parcels of flowers which reach hospitals are as often sources of disappointment as of pleasure. Many a sister and nurse bent upon beautifying her ward and giving delight to those about her, receives the basket with joy and opens it with fingers tremulous with anticipation. Exclamations of rapture greet the well-preserved contents, but when a glance reveals, as it too often does, that the flowers have mostly drooped beyond recovery, or have tumbled to pieces, the disappointment is keen.

We have witnessed this disappointment many a time, and it seems a pity that a happier and less wasteful result is not assured. We must, however, begin at the beginning; perhaps it may be said not inaptly, we must go to the root of the matter. For it is certain that many gardens are entirely unprovided with flowers fitted for indoor decoration, and those who wish for a succession of suitable blossoms for their own homes and for gifts to hospitals must work upon a plan differing altogether from the three months, "bedding out" system which too often takes the place of real gardening.

Visitors to one of the exhibitions held during recent years at South Kensington will remember the display of flowers made during the whole period of the exhibition by a well-known florist whose gardens are situated in the outskirts of the metropolis, and who from out a somewhat grimy neighbourhood supplied day by day consignments of blossoms notable alike for size and colour, a very revelation of beauty undreamed of even by many of those who claimed familiarity with things horticultural. An hour's study of that marvellous and ever changeful group of hardy flowers was in itself a floral education. It showed the varied splendour our gardens may be made capable of, even under conditions not wholly advantageous, and doubtless it exercised something more than a passing influence on perennial flower-borders. If so, both hospitals and housewives will benefit. The home is often destitute of flowers simply because the garden does not produce the sorts wanted. Every well-managed garden ought to supply the table and the sideboard for eight or nine months of the year, and with the many whose interest is almost wholly in cut flowers, neither satisfaction nor encouragement is felt when the flowers are too flimsy to come successfully through the perils of arrangement or too unwieldy to hold up their heads to admiration.

In regard to gifts of flowers to hospitals, we may reflect that a very tender and even a religious sentiment is often pourtrayed in an offering of this nature. It is not given to everyone, no matter how great his compassion, to work actively for the sick. Yet many who would instinctively shrink from actual contact with scenes of suffering and who would be out of place in a sick room, so far from being indifferent, may be endowed with a force of sympathy which only needs direction to expend itself in deeds of love and usefulness. And though the token of their interest be nothing more than a gift of commonplace flowers, let us picture them as true benefactors bent upon doing their share of the great work which everyone ought to hold in sympathising remembrance. Givers actuated by these feelings would certainly, though perhaps unconsciously, prove themselves subject to the high ethical law which demands of us in all we undertake the exercise of our highest powers. They would desire their gifts should be of the best, that is the most suitable, the most likely to give pleasure and to repay anticipation by service not too ephemeral.

This sentiment might be practically illustrated if those possessed of enough ground would set apart some sunny corner for an "hospital garden." In it might be grown such plants as answer to the conditions indicated. We have seen that variety is assured: there need be no fear about extravagance. It is the "bedding out" gardening which is costly and wasteful. Nothing but indolence or ignorance brings about the surrender of our gardens to treatment which wakes them to feverish life during a few fleeting weeks of summer, and then while the flush is still upon them, lets them die as if of shame at their own native barrenness.

The arrangement of our hospital garden may be simplicity itself, but it should be tasteful simplicity. While it cannot be pretended that the blossoms when cut will be less lovely because the plants which bore them stood in rows, like cabbages, yet we think the garden ought to be comely and attractive—a thing of veriest beauty, in the fashioning of which we have put the best work of which we are capable—and that the pleasure of tending it will be greater, and the legends which in time will twine themselves about its borders will be more tender if the surroundings be graceful. And only consider of what woof these legends will be woven; how every thread will be hung with memories of smiles brought to wan faces, and little episodes of glad welcome. We send them forth—these blossoms—as ambassadors from the world of health and sunny life and freshest beauty, charged with messages of sympathy to those who lie in suffering and sorrow, bidding them be of good cheer for the love and pity we bear them.

Think of the pleasant musings we may have when we linger among the borders where every bloom is harvested for Charity's sheaf, and the air is so full of sweetness that our thoughts become fragrant as the flowers. Many lessons will be taught there, and most of all shall we learn thankfulness, when we think upon the feebleness and dependence of those poor folk passing through the dark valley, and that we, still standing in the sunshine, must bestir ourselves if we would not have to remember when we are laid low that, as we moved in the brightness of life's summer, we did no more than cast a shadow upon the world about us.

And if the disposition of our garden may be simple, the variety of flowers grown need not be so great as to be perplexing. Comparatively a few plants of the right sorts will go far. In our ideal hospital garden we should like to have Glory of the Snow (Chinodoxa), hyacinths, tulips, narcissus, especially polyanthus varieties, the double white (alba plena odorata), and pheasant's eye, wallflowers, campanulas, iris, gladioli, early (Nanus), intermediate (Ramosus), and late (Brenchleyensis and Grandavensis), roses, lilies of various kinds, particularly the June red (umbellatum), the white trumpet Bermuda lily (longiflorum), the Madonna (candidum), the tiger, and lancifolium varieties; the golden lily of Japan (L. auratum), is magnificent in the garden, but a little too strongly scented for a sick room—pyrethrums, including uliginosum, which flowers freely in October, delphiniums, foxgloves, carnations, geums, spireas, including filipendula, lychnis, notably chalcedonica, gaillardias, phloxes, Iceland poppies, helianthus, dahlias, tritomas, hollyhocks, rudbeckias, asters, and chrysanthemums, early and late varieties. Of annuals, we should choose sweet peas, marigolds of all kinds, stocks, larkspurs, asters, zinnias, chrysanthemum (coronarium), everlastings (helichrysum), and, of course, mignonette.

The last we should grow for its sweetness; the rest fulfil the conditions laid down; they bear showy and long-abiding blossoms, and together they will maintain a supply of flowers from March to November. Of the perennials, dahlias and gladioli alone need be moved from the open ground, where the remainder will flourish freely, and in numerous instances the roots may be divided year by year. The single and double perennial sunflowers (harpalium rigidum, helianthus multiflorus plenus, hel. major, etc.), yield splendid flowers for large rooms; so do the tritomas (red-hot pokers). A dozen well-grown plants of helianthus, and the same number of common double, single, and cactus dahlias, will provide a succession of magnificent blossoms during part of July, August, September, and perhaps October, enough to keep several hospital wards well supplied. Gaillardias alone will give grand combinations of crimsons and yellows for four entire months, beginning in June, and the gladioli, perhaps the most superb of all flowers, may be spread over an equally long period.

In the depth of winter, cuttings from green and variegated shrubs form a charming setting for the handsome blossoms of helichrysum, which ought to have been gathered before they were fully opened, and put away in boxes when dry. These beautiful flowers are produced in abundance from July until the frost cuts them down, and, although the plants then perish utterly like other annuals, their blossoms, if they have been carefully husbanded, remain to lend rich colour to the dull period of mid-winter, when flowers in the open borders are, and in our climate must ever remain, rarities.

Those who have the opportunity may appropriately give fruit a place in their hospital garden. Strawberries and pears will be the most serviceable sorts for the open ground. If there be a sunny wall, peaches and nectarines are possibilities; but in England the cultivation of wall fruit is prolific of disappointment, and it has been recently said that it is becoming a lost art. To those ambitious to provide more than flowers, grapes will suggest themselves at once. A small house furnished with two or three "Black Hambro'" or "Buckland's Sweet Water" vines, needing nothing beyond a little attention and no artificial heat, will produce scores of pounds of the fruit, which, of all kinds, is most in demand for sick people, and which has this distinct advantage over other soft fruits: it need not be gathered in a hurry and eaten directly it is ripe, but, if carefully husbanded, will provide a harvest of two month's duration at least, while in ordinary circumstances, vines are of so regular habit that almost every year is a year of plenty.

B. Burford Rawlings.

